# Arp2/3 Complex Inhibition Prevents Meiotic Maturation in Porcine Oocytes

**DOI:** 10.1371/journal.pone.0087700

**Published:** 2014-01-31

**Authors:** Fei Wang, Ga-Young An, Yu Zhang, Hong-Lin Liu, Xiang-Shun Cui, Nam-Hyung Kim, Shao-Chen Sun

**Affiliations:** 1 College of Animal Sciences and Technology, Nanjing Agricultural University, Nanjing, China; 2 Department of Animal Sciences, Chungbuk National University, Cheongju, Korea; 3 College of Veterinary Medicine, Nanjing Agricultural University, Nanjing, China; Institute of Zoology, Chinese Academy of Sciences, China

## Abstract

The Arp2/3 complex regulates actin nucleation, which is critical for a wide range of cellular processes, such as cell polarity, cell locomotion, and endocytosis. In the present study, we investigated the possible roles of the Arp2/3 complex in porcine oocytes during meiotic maturation. Immunofluorescent staining showed the Arp2/3 complex to localize mainly to the cortex of porcine oocytes, colocalizing with actin. Treatment with an Arp2/3 complex specific inhibitor, CK666, resulted in a decrease in Arp2/3 complex localization at the oocyte cortex. The maturation rate of porcine oocytes decreased significantly after CK666 treatment, concomitant with the failure of cumulus cell expansion and oocyte polar body extrusion. The fluorescence intensity of F-actin decreased in the cytoplasm, and CK666 also disrupted actin cap formation. In summary, our results illustrate that the Arp2/3 complex is required for the meiotic maturation of porcine oocytes and that actin nucleation is critical for meiotic maturation.

## Introduction

In mammals, oocytes undergo maturation before fertilization. When mammalian follicles grow, granulosa cells proliferate and differentiate into two types of cells, namely cumulus cells, which encircle the growing oocyte many times and mural granulosa cells, which comprise the innermost layer of the follicle wall. During the pre-ovulatory phase, cumulus cells undergo a series of transformations defined as cumulus expansion, which is essential for fertilization [1]. Cumulus expansion also plays a key role in disseminating local and endocrine signals to oocytes [2]. Furthermore, cumulus expansion is required for ovulation and the subsequent development of the zygote [3], [4]. On the other hand, fully-grown oocytes are arrested at the germinal vesicle (GV) stage in mature ovarian follicles, and they resume meiosis only after being released from the follicle. After germinal vesicle breakdown (GVBD), spindles form as chromatin condenses and microtubules reorganize. The oocytes enter metaphase I (MI), followed by peripheral spindle migration and first polar body extrusion. The oocytes then enter metaphase II (MII) and arrest at this stage until fertilization. During oocyte maturation in mice, the spindle moves from a central position to the cortex, resulting in the small polar body extrusion [5]–[7].

Actin is involved in several processes, such as cell morphology, cytokinesis, and cell movement. Actin also has important roles in mammalian oocyte maturation. After GVBD, actin surrounds the GV and facilitates chromosome congression [8]. After spindle formation, however, the centrally-formed spindle migrates and anchors onto the cortex in an actin-dependent manner [9], a process that involves dynamic actin changes [10]. In addition, actin associates with chromosomes and is enriched at the site of the actin cap [11]. Lastly, actin, together with myosin, facilitate the formation of a contractile ring and promote first polar body extrusion [12].

The Arp2/3 complex (actin-related protein 2/3 complex) is comprised of Arp2, Arp3, and Arpc1 to Arpc5 (five individual subunits) [13], [14]. Arp2 and Arp3 are actin-related proteins that are linked by the different Arpc subunits to the mother filament, and they nucleate the growth of new actin filaments [15]. The Arp2/3 complex participates in a wide range of cellular processes, including cell migration and adhesion [16], endocytosis [17], and cell polarity during mitosis [15]. The Arp2/3 complex also interacts with nucleation-promoting factors (NPFs) to mediate branched-actin network formation, which is required for cytoskeletal remodeling, intracellular transport, and cell locomotion [18].

Although the Arp2/3 complex plays critical roles in many cell types, there is no report on the involvement of the Arp2/3 complex in porcine oocytes during meiotic maturation. This study aimed to investigate the effects of the Arp2/3 complex on porcine oocyte meiotic maturation by a specific inhibitor CK666. Our results illustrate that the Arp2/3 complex is essential for the meiotic maturation of porcine oocytes and that actin nucleation is important for meiotic maturation.

## Materials and Methods

### Ethics statement

Animals use and care were in accordance with Animal Research Institute Committee guidelines prescribed by Nanjing Agricultural University, China. Ovaries were obtained from 6 month-old Duroc gilts at the Nanjing Tianhuan Food Corporation slaughterhouse (Nanjing, China) and transported to the laboratory at 25°C in Dulbecco's phosphate-buffered saline (dPBS). This study was specifically approved by the Committee of Animal Research Institute, Nanjing Agricultural University, China. And the permission was obtained from the Nanjing Tianhuan Food Corporation slaughterhouse to use these animal parts.

### Antibodies and chemicals

The mouse monoclonal anti-ARP2 antibody (ab49674) was purchased from Abcam (Cambridge, UK), and CK-666 was obtained from Merck (Darmstadt, Germany). The mouse monoclonal anti-α-tubulin antibody (F2168) and phalloidin-TRITC (P1951) were purchased from Sigma-Aldrich (St. Louis, MO, USA). The Alexa Fluor 488 goat anti-mouse secondary antibody (A11001) was purchased from Invitrogen (Carlsbad, CA, USA).

### Oocyte collection, culture, and treatment

Cumulus-oocyte complexes (COCs) were aspirated from antral follicles (3–6 mm in diameter). Only COCs with multiple layers of intact cumulus cells and a uniform ooplasm were selected for in vitro maturation (IVM). Approximately 50 COCs were matured in 200 µl of IVM medium (containing with 0.1% (wt/vol) polyvinyl alcohol (PVA; Sigma-Aldrich Co., USA), 32.5 mM sodium bicarbonate, 0.91 mM sodium pyruvate, 3.05 mM glucose, 75 mg/L penicillin and 50 mg/L streptomycin, respectively) under mineral oil at 38.5°C for 24 h [for COCs at metaphase I (MI)] or 44 h [for COCs at metaphase II (MII)] in a humidified atmosphere of 5% CO_2_ (v/v). After maturation, cumulus cells were removed by pipetting in the presence of 0.1% hyaluronidase (w/v) for 2 min. CK666 (50 mM in DMSO) was diluted in TCM-199 medium to a final concentration of 100 µM or 250 µM. The control group was treated with an identical concentration of DMSO.

### Confocal microscopy

Oocytes were fixed in PBS containing 4% paraformaldehyde (w/v) at room temperature (R.T.) for 30 min and then transferred to permeabilization solution (1% Triton X-100, v/v) for 8 h at 37°C. After blocking for 1 h in PBS supplemented with 1% BSA (w/v), oocytes were incubated for 4 h at R.T. with the anti-ARP2 antibody (1∶200) and phalloidin-TRITC (5 µg/ml). After washing three times in washing buffer (0.1% Tween 20 in PBS, v/v), oocytes were incubated for 1 h at R.T. with Alexa Fluor 488 goat anti-mouse IgG (1∶100, to detect ARP2 staining). Oocytes were stained with Hoechst 33342 for 10 min, washed three times in washing buffer, mounted onto slides, and examined with a confocal laser scanning microscope (Zeiss LSM700 META). At least 30 oocytes were examined for each group.

### Data analysis

Statistical analysis was conducted using analysis of variance (ANOVA), and differences between treatment groups were assessed by Duncan's multiple comparison test. Data were from at least three replicates and p<0.05 was considered statistically significant. Fluorescence intensities were quantified by Image J software (NIH, USA), and ten oocytes were analyzed for each experiment.

## Results

### Localization of the ARP2/3 complex in porcine oocytes during meiotic maturation

**Figure 1:**
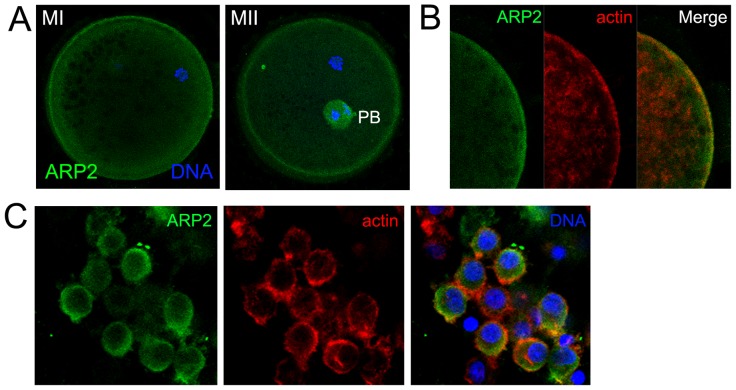
Localization of ARP2 and actin in porcine oocytes during meiotic maturation. (**A**) Subcellular localization of the Arp2/3 complex in porcine oocytes during meiotic maturation. An anti-ARP2 antibody was used to detect the subcellular localization of the Arp2/3 complex. ARP2 accumulated at the cortex and in the cytoplasm of MI and MII oocytes. ARP2, green; chromatin, blue. Bar = 20 µm. (**B**) Subcellular localization of ARP2 and actin in porcine oocytes during meiotic maturation. A similar localization pattern was observed for ARP2 and actin in porcine oocytes. ARP2, green; actin, red; chromatin, blue. (**C**) Subcellular localization of ARP2 and actin in porcine cumulus cells. ARP2, green; actin, red; chromatin, blue. Bar = 20 µm.

Using immunofluorescent staining, we examined the subcellular localization of the Arp2/3 complex. As shown in Fig. 1A, ARP2 was enriched in the cytoplasm and localized mainly at the cortex of MI and MII oocytes. To further confirm this finding, we co-stained ARP2 and actin (Fig. 1B). ARP2 colocalized with actin. We also stained ARP2 in porcine cumulus cells. ARP2 localized to the cytoplasm and the cortex of cumulus cells (Fig. 1C)

### CK666 treatment decreases the expression of the Arp2/3 complex in porcine oocytes

**Figure 2:**
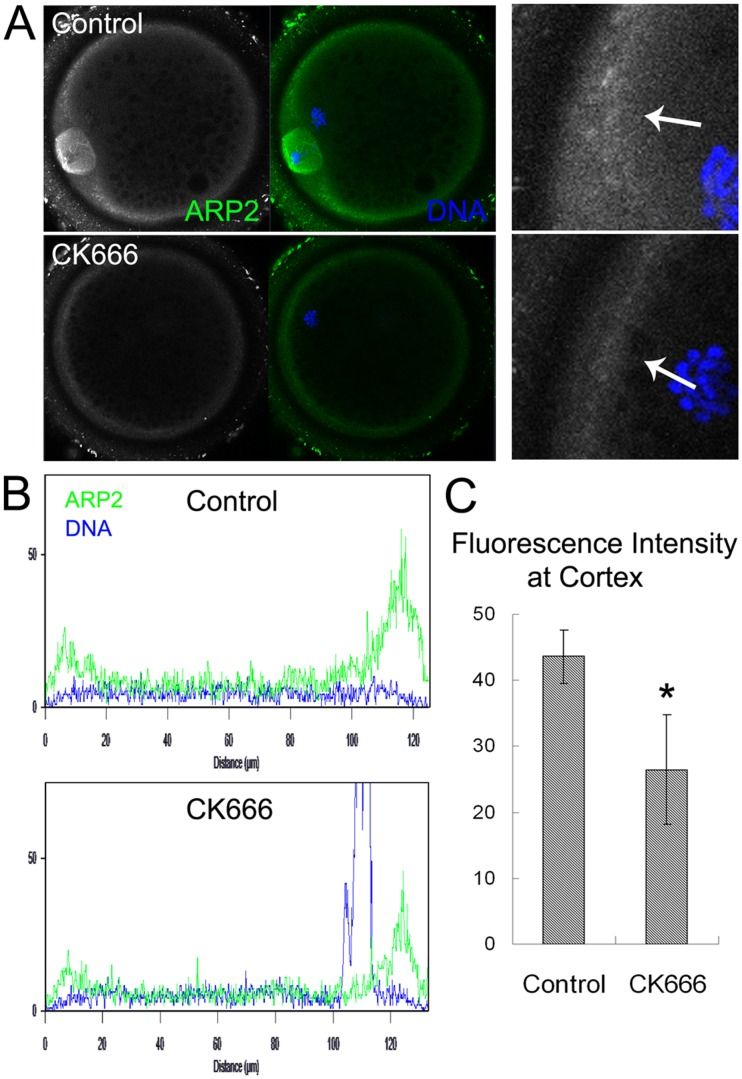
CK666 treatment inhibits the nucleation of the Arp2/3 complex in porcine oocytes. (**A**) The expression of ARP2 after treatment with CK666. (**B**) The fluorescence intensity of ARP2 decreased significantly in oocytes treated with CK666. (**C**) Measurement of ARP2 fluorescence intensity after CK666 treatment *, p<0.05.

We employed CK666, an Arp2/3 complex specific inhibitor, to investigate the function of the Arp2/3 complex in porcine oocytes during meiotic maturation. We first examined the effect of CK666 on Arp2/3 complex expression. After culturing oocytes for 48 h in medium containing CK666, the expression of ARP2 decreased significantly at the cortex (Fig. 2A). To confirm this finding, we examined the fluorescence intensity of ARP2 in oocytes. The fluorescence intensity of ARP2 in CK666-treated oocytes was lower than that of control oocytes (Fig. 2B). Measurement of the ARP2 fluorescence intensity (43.6±4.1 for control oocytes vs. 26.5±8.3 for CK666-treated oocytes, n = 30) (p<0.05) confirmed these results (Fig. 2C).

### CK666 treatment decreases the meiotic maturation of porcine oocytes

**Figure 3:**
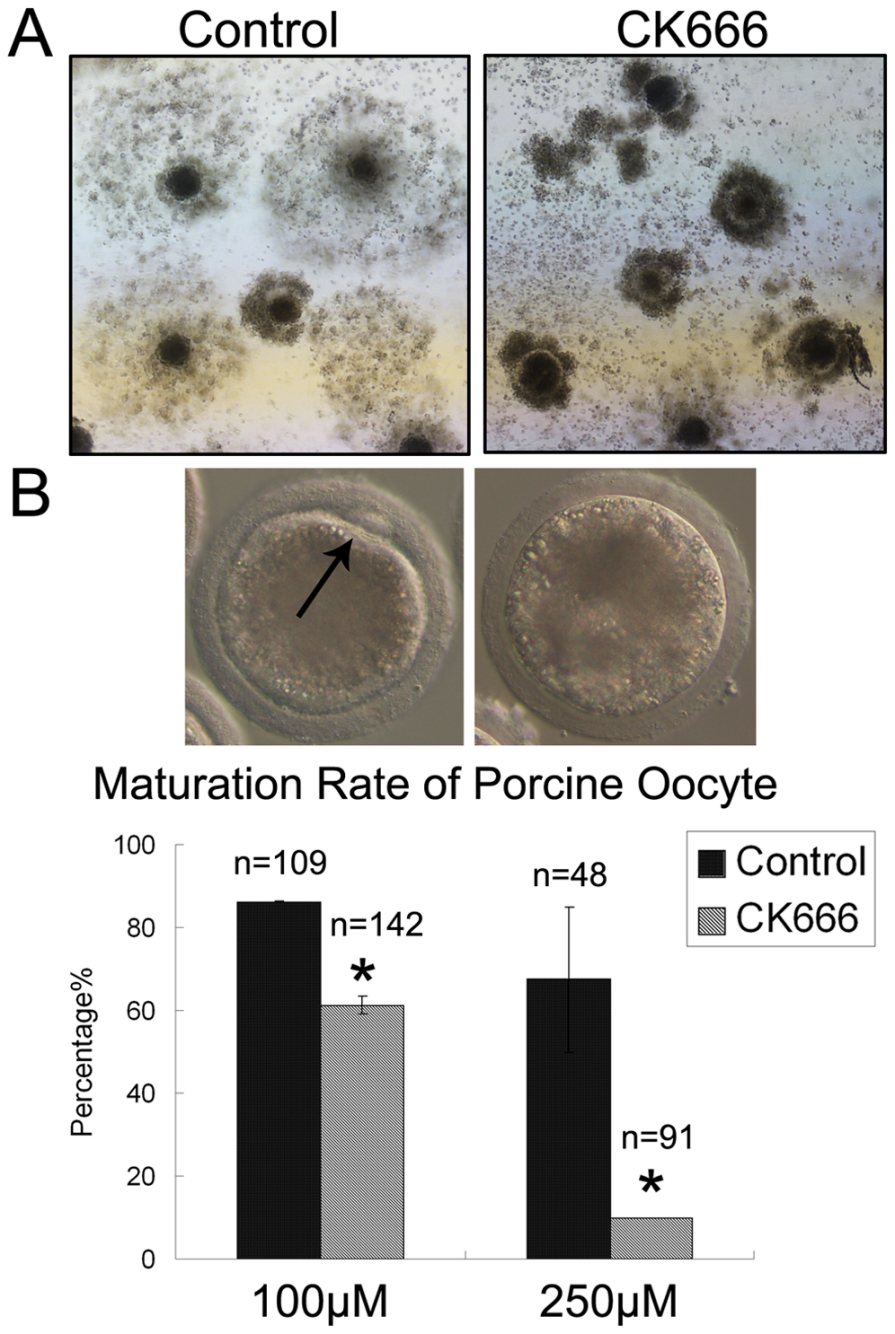
CK666 treatment inhibits the maturation of porcine oocytes. (**A**) Cumulus cell expansion failed to occur after treatment with CK666. (**B**) The oocyte maturation rate decreased significantly after treatment with CK666. *, p<0.05.

We next examined the effects of CK666 on porcine oocyte maturation. Cumulus expansion is important for ovulation, fertilization, zygote development, and it is an indicator of oocyte maturation. It also regulates meiosis and supports the cytoplasmic maturation of oocytes. After culturing COCs for 48 h in medium containing 100 µM CK666, cumulus expansion did not occur in oocytes (Fig. 3A). Furthermore, CK666 treatment inhibited the meiotic maturation of porcine oocytes, and the inhibition was dose-dependent [86.2±0.2% (n = 109) for control oocytes vs. 61.2±2.2% (n = 142) for oocytes treated with 100 µM CK666 and 67.5±17.5% (n = 48) for control oocytes vs. 9.9±0.1% (n = 91) for oocytes treated with 250 µM CK666] (p<0.05) (Fig. 3B).

### CK666 treatment disrupts actin expression

**Figure 4:**
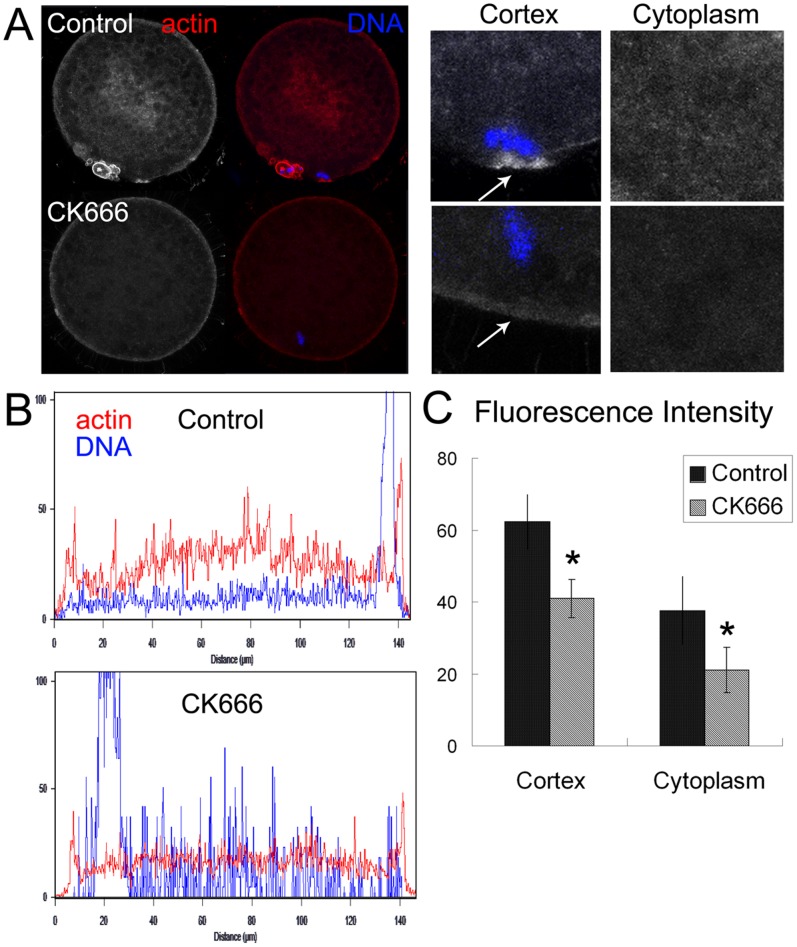
CK666 treatment decreases actin nucleation. (**A**) An actin cap formed at the cortex of a control oocyte. There was no actin cap formation, and actin nucleation at the plasma membrane and in the cytoplasm decreased after CK666 treatment. Actin, red; chromatin, blue. Bar = 20 µm. (**B**) The fluorescence intensity of actin decreased significantly in oocytes cultured with CK666. (**C**) Measurement of actin fluorescence intensity after CK666 treatment *, p<0.05 both at the cortex and in the cytoplasm of oocytes.

The Arp2/3 complex is involved in actin assembly in many different cell types. To investigate the mechanism behind the failure of porcine oocytes to mature after CK666 treatment, we examined actin expression. After culturing oocytes for 48 h, polar body extrusion was observed, followed by the arrest of oocytes at MII. Actin also localized to the cortex, and actin cap formation was observed at the site of chromosomes. After culturing oocytes for 48 h in medium containing 100 µM CK666, actin expression decreased significantly both at the cortex and in the cytoplasm. Moreover, the actin cap also disappeared after CK666 treatment (Fig. 4A). The fluorescence intensity of actin both at the cortex and in the cytoplasm of CK666-treated oocytes decreased significantly compared to the control group (Fig. 4B). Measurement of the actin cap fluorescence intensity [62.3±7.4 vs. 40.9±5.3 for the cortex and 37.6±9.4 vs. 21.1±6.4 for the cytoplasm, (n = 30)] (p<0.05) confirmed these results (Fig. 4C).

## Discussion

In the present study, we investigated whether the Arp2/3 complex is involved in the meiotic maturation of porcine oocytes. Disruption of Arp2/3 complex activity by a specific inhibitor, CK666, inhibited actin expression at the cortex and in the cytoplasm of oocytes, as well as reducing the meiotic maturation rate. These results illustrate that the Arp2/3 complex is involved in the meiotic maturation of porcine oocytes through the regulation of actin assembly.

During the meiotic maturation of porcine oocytes, the Arp2/3 complex was enriched at the cortex, colocalizing with actin. This localization pattern is similar to previous studies that described Arp2/3 complex localization at the plasma membrane together with actin in somatic cells or embryo fibroblasts from other species [19]–[22]. This localization pattern indicates that the Arp2/3 complex may have a conserved role that is related to the function of actin and that the Arp2/3 complex may be involved in actin-related processes during meiotic maturation in porcine oocytes.

To test our hypothesis, we inhibited the Arp2/3 complex by CK666 treatment. During ovulation, cumulus cells surrounding the oocyte secrete hyaluronan (HA). HA accumulates in cumulus cells and embeds them in a gelatinous matrix. This process, defined as cumulus expansion [23], [24], regulates meiosis and supports the cytoplasmic maturation of oocytes. Our results show that after inhibition of the Arp2/3 complex by CK666 treatment, oocyte maturation failed, which was characterized by the absence of cumulus expansion and oocyte polar body extrusion. Previous studies have shown that activators of the Arp2/3 complex, WAVE2 and JMY, are necessary for polar body extrusion during the meiotic maturation of mouse oocytes [25], [26]. Our results are consistent with these reports, and they show that the Arp2/3 complex has a conservative role for mammalian oocyte meiotic maturation.

We also investigated the mechanism behind the role of the Arp2/3 complex in the meiotic maturation of porcine oocytes. During mitosis, the Arp2/3 complex and NPFs mediate the assembly of branched-actin filament networks, which are required for phagocytosis and lamellipodia formation [13], [27]. Thus, a decrease in actin expression after CK666 treatment may result in the failure of oocyte maturation. Our results also illustrate that inhibition of Arp2/3 complex activity resulted in the disruption of actin cap formation, as well as in the degradation of actin both at the cortex and in the cytoplasm of porcine oocytes. The observations are indicative of cytokinesis defects, and they support the contention that actin is critical for cell division, especially for the contractile ring formation. Actin also regulates polar body extrusion in mouse oocytes, and several molecules play an important role in cytokinesis via their effects on the actin cytoskeleton. For example, Cdc42 and Rac, which activate Arp2/3, are necessary for polar body extrusion and spindle migration during meiotic maturation [28]–[30]. Ran GTPase, which functions upstream of the Arp2/3 complex, also plays an important role in controlling cortical polarity during polar body extrusion in mouse oocytes by mediating chromatin signaling [31]. Our results show that the regulatory mechanism for the Arp2/3 complex in porcine oocytes was also mediated through an actin-dependent pathway.

In conclusion, our results indicate that the Arp2/3 complex regulates meiotic maturation in porcine oocytes by facilitating cumulus expansion and actin nucleation.
